# Morphologies Tuning of Polypyrrole and Thermoelectric Properties of Polypyrrole Nanowire/Graphene Composites

**DOI:** 10.3390/polym10101143

**Published:** 2018-10-13

**Authors:** Yong Du, Hao Niu, Jun Li, Yunchen Dou, Shirley Z. Shen, Runping Jia, Jiayue Xu

**Affiliations:** 1 School of Materials Science and Engineering, Shanghai Institute of Technology, 100 Haiquan Road, Shanghai 201418, China; nh954779551@163.com (H.N.); 156081106@mail.sit.edu.cn (J.L.); yunchendou@sit.edu.cn (Y.D.); Jiarp@sit.edu.cn (R.J.); 2 CSIRO Manufacturing, Private Bag 10, Clayton South, VIC 3169, Australia; shirley.shen@csiro.au

**Keywords:** polypyrrole nanowire, graphene, composite, thermoelectric

## Abstract

Polypyrrole (PPy) with different morphologies (e.g., particles, nanotubes, and nanowires) were successfully prepared by adding or without adding different kinds of surfactants through a chemical oxidative polymerization method, respectively. The results show that the morphologies of PPy can be effectively controlled and have a significantly effects on their thermoelectric properties. The PPy nanowires exhibit the highest electrical conductivity and Seebeck coefficient among the various PPy morphologies, such as particles, nanotubes, and nanowires, so PPy nanowires were chosen to prepare PPy nanowire/graphene thermoelectric composites via a soft template polymerization method using cetyltrimethyl ammonium bromide as the template. Both electrical conductivity and Seebeck coefficient of the PPy nanowire/graphene composites increased as the content of graphene increases from 0 to 20 wt %, and as the measured temperature increases from 300 K to 380 K, which leds to the same trend for the power factor. A highest power factor of 1.01 μWm^−1^K^−2^ at ~380 K was obtained for the PPy nanowire/graphene composites with 20 wt % PPy nanowire, which is about 3.3 times higher than that of the pure PPy nanowire.

## 1. Introduction

Since the discovery of the Seebeck effect in 1821 by German scientist Thomas Johann Seebeck [1], most of researchers have focused on inorganic thermoelectric (TE) materials, such as Bi-Te-, Pb-Te-, and Si-Ge-based alloys, and this phenomenon has continued until the begining of the 21st century. More and more attention has been paid to conducing polymer TE materials and their corresponding TE nanocomposite materials [2,3,4,5].

Polypyrrole (PPy), one kind of conducting polymers, has many outstanding features, such as, low density, low thermal conductivity, and easy fabrication [3,6]; therefore, PPy has a great potential for TE materials individually or as a matrix for nanocomposites. A PPy film was prepared by an electrochemical method, and a power factor (*PF* = *S*^2^*σ*, where *S* is Seebeck coefficient and *σ* is electrical conductivity) of 3.9 μWm^−1^K^−2^ (*ZT* value of 6.8 × 10^−3^) was achieved at 300 K [6]. A lamellar PPy doped with β-naphthalene sulfonic acid was fabricated, and a power factor of about 0.4 μWm^−1^K^−2^ (*ZT* value of 6.2 × 10^−3^) was obtained with the molar ratio of monomer pyrrole to β-naphthalene sulfonic acid being 1.0:0.45 at 300 K [7]. In addition, a multi-walled carbon nanotube (MWCNT)/PPy composite was fabricated by an in situ polymerization method, and a power factor of 2.2 μWm^−1^K^−2^ was achieved for the composite with 68 wt % MWCNT at room temperature (RT) [8]. A single-walled carbon nanotube (SWCNT)/PPy nanocomposite was made by an in situ polymerization method, and a power factor of 5.46 μWm^−1^K^−2^ was obtained at 398 K for the nanocomposite with 40 wt % SWCNT [9]. A reduced graphene oxide (rGO) nanosheet/PPy composite was prepared by a template-directed in situ polymerization, and a power factor of 3.01 μWm^−1^K^−2^ was achieved with the rGO/Py mass ration of 2:1 at RT [10].

Poly(3,4-ethylenedioxythiophene) (PEDOT), one of the most studied conducing polymers, shows a potential application prospect in TE [11]. The morphologies of PEDOT have significantly effects on its TE properties, as both the Seebeck coefficient and electrical conductivity of PEDOT were reported increased with the morphologies of globular nanoparticles, nanotubes, and nanofibers [12]. Wu et al. [13] reported that the p-toluenesulfonic acid (p-TSA) doped polyaniline (PANi) nanowires showed higher Seebeck coefficient and lower thermal conductivity than that of p-TSA-doped nanorods. However, most of the research on PPy were mainly focused on PPy particles (PPy-PTs) and PPy-PT-based TE nanocomposites. The research on the 1-D PPy nanostructures, e.g., PPy nanotubes (PPy-NTs), PPy nanowires (PPy-NWs), and their corresponding TE composites is very limited, although some progress have been reported [14,15]. A polypyrrole nanotube (PPy-NT) film was prepared, and a power factor of 0.31 μWm^−1^K^−2^ (*ZT* value of 5.71 × 10^−4^) at 310 K was obtained [14]. A PPy-NWs was prepared and a power factor of 22.6 × 10^−3^ μWm^−1^K^−2^ was achieved [15]. It is a significant need to investigate the morphologies of PPy (e.g., PPy-PTs, PPy-NTs, and PPy-NWs) on the TE properties of themselves and their corresponding composites.

Graphene, is commonly used as a filler for the conducting polymer matrix to prepare conducting polymer based nanocomposites [16]. For example, PPy/graphene nanocomposite can be used in many areas [17,18,19,20,21], such as, lithium batteries [19], supercapacitors [20], electrode materials [21], etc. Furthermore, as a kind of fillers, graphene can also improve the TE properties of the conducting polymers, due to its high electrical conductivity [22,23]. For example, the graphene/poly(3,4-ethylenedioxythiophene) (PEDOT) composites [23] and graphene/polyaniline (PANI) composites [22] were reported. However, so far, to the best of our knowledge, no systematic research about the influence of morphologies of PPy (e.g., PPy-PTs, PPy-NTs and PPy-NWs) on their TE properties and PPy-NW/graphene composites has been reported. In this work, PPy with different morphologies (PPy-PTs, PPy-NTs and PPy-NWs) were successfully prepared by adding or without adding different kinds of surfactants through a chemical oxidative polymerization method, respectively. The effects of the morphologies of PPy-PTs, PPy-NTs, and PPy-NWs on their TE properties have been investigated, and the results show that PPy-NWs has the highest power factor. Therefore, PPy-NW/graphene TE composites have been prepared via a soft template polymerization method using cetyltrimethyl ammonium bromide as the template. The compositions, morphologies, and TE properties of the PPy-NW/graphene composites with different contents of graphene have also been investigated.

## 2. Experimental

### 2.1. Materials

Pyrrole monomer (chemical grade) was purchased from Sigma-Aldrich (Shanghai, China). Methyl orange (reagent grade) and cetyltrimethyl ammonium bromide (CTAB, reagent grade) were purchased from Adamas Reagent Co., Ltd. (Shanghai, China). FeCl_3_·6H_2_O (analytical reagent), and absolute ethanol (reagent grade) were purchased from Sinopharm Chemical Reagent Co., Ltd. (Shanghai, China). Ammonium peroxydisulfate (APS) (oxidant) was purchased from Shanghai Titanchem Co., Ltd. (Shanghai, China) Graphene was purchased from Nanjing XFNANO Materials Tech Co., Ltd. (Nanjing, China). All the materials were used without further treatment or purification.

### 2.2. Preparation of the PPy Particles

Typical synthetic process of PPy particles was as follow [24]: 0.55 mL of pyrrole monomer was dissolved in 100 mL of deionized water (Solution A). 8.65 g of FeCl_3_·6H_2_O was dissolved in 100 mL of deionized water (Solution B), which was added to Solution A to initiate the polymerization. The mixed solution was constantly stirred at 50 rpm for 8 h at RT. The products was washed with deionized water and absolute ethanol successively for three times, and then dried at 60 °C for 12 h.

### 2.3. Preparation of the PPy Nanotubes

Typical synthetic process of PPy nanotubes was as follow [25]: 0.49 g methyl orange (MO) and 0.56 mL pyrrole monomer was dissolved in 150 mL of deionized water (Solution A). Then 8.72 g FeCl_3_·6H_2_O dissolved in 100 mL of deionized water (Solution B), was then added to Solution A and constantly stirred for 8 h at RT. The products was washed with deionized water and absolute ethanol successively for three times, and then dried at 60 °C for 12 h.

### 2.4. Preparation of the PPy-NWs and PPy-NW/Graphene Nanocomposites

**Figure 1:**
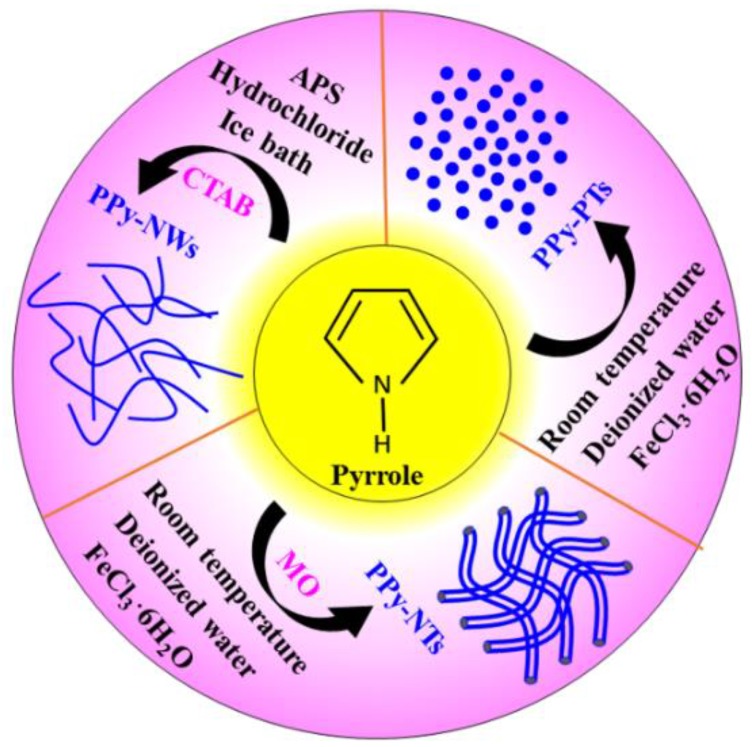
Schematic illustration of the formation procedure of PPy-PTs, PPy-NTs, and PPy-NWs.

**Figure 2:**
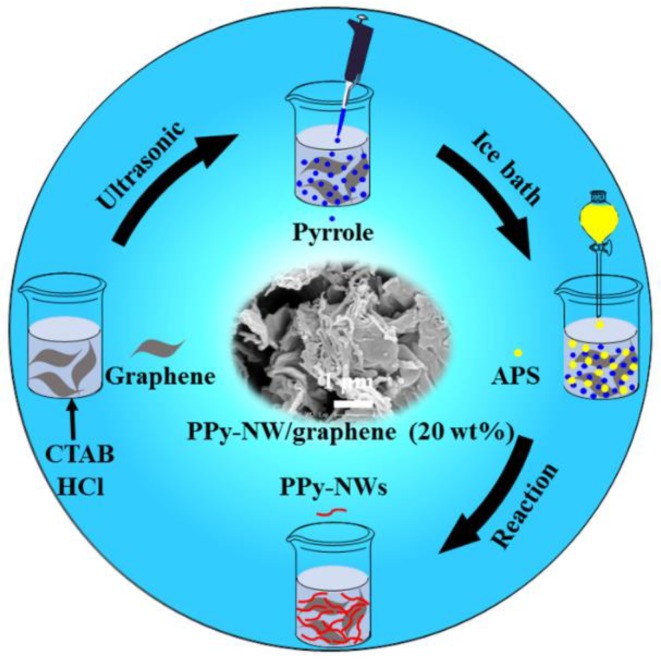
Schematic illustration of the procedure for preparation of PPy-NW/graphene composites.

Typical synthetic process of PPy-NWs was: 0.66 g CTAB was dissolved in 200 mL of 0.5 mol/L hydrochloride (HCl) (Solution A), a designed mount of graphene (5 wt %, 10 wt %, 15 wt %, and 20 wt %) was added into Solution A, stirred for 30 min and then ultrasoniced for 2 h. 1.6 mL pyrrole monomer was added into the above solution to form Solution B. 1.23 g APS dissolved in 200 mL of 0.5 mol/L HCl (Solution C), was then added to Solution B and constantly stirred for 8 h in an ice bath. The products was washed with deionized water and absolute ethanol successively for three times, and then dried at 60 °C for 8 h. For a comparison, PPy-NWs were also prepared using the same procedure but without graphene. Figure 1 illustrates the formation procedure of PPy-PTs, PPy-NTs, and PPy-NWs. Figure 2 illustrates the procedure for the preparation of PPy-NW/graphene composites.

### 2.5. Characterizations

The phase composition and morphology of the samples were characterized using X-Ray photoelectron spectroscopic (XPS, PHI 5000 VersaProbe, ULVAC-PHI, Chigasaki, Japan), scanning electron microscopy (SEM, Philips XL 30 FEG, Philips, Eindhoven, The Netherlands), and transmission electron microscopy (TEM, CM200-FEG, Philips, Eindhoven, The Netherlands), respectively. The electrical conductivity and Seebeck coefficient were measured simultaneously in a vacuum atmosphere from 300 to 380 K on an MRS-3L thin-film thermoelectric test system (Wuhan Giant Instrument Technology Co., Ltd., Wuhan, China).

## 3. Results and Discussion

**Figure 3:**
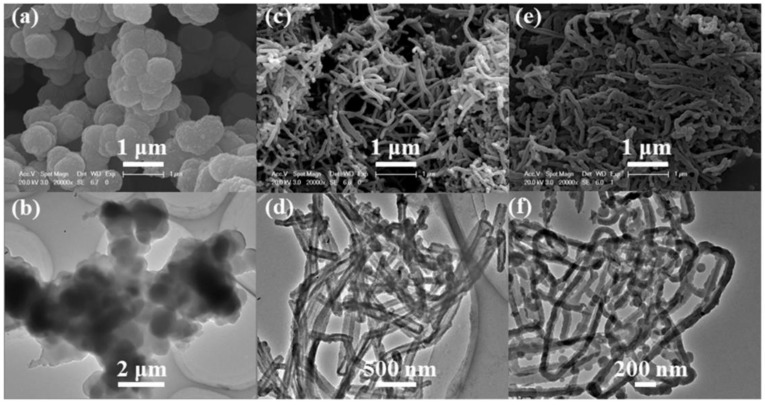
SEM images of (**a**) PPy-PTs, (**c**) PPy-NTs, and (**e**) PPy-NWs. TEM images of (**b**) PPy-PTs, (**d**) PPy-NTs, and (**f**) PPy-NWs.

Figure 3 shows the SEM and TEM images of the PPy-PTs, PPy-NTs, and PPy-NWs, respectively. When FeCl_3_·6H_2_O was used as an oxidant without a surfactant, PPy-PTs were obtained and agglomerated together to form an analogous spherical morphology (Figure 3a,b), which agrees with the Reference [9]. When FeCl_3_·6H_2_O and MO were used as an oxidant and a surfactant, respectively, PPy-NTs were achieved (Figure 3c,d), mainly due to pyrrole monomer was polymerized on the surface of the 1D templates [25,26,27], which were formed due to MO change from its salt form (high water solubility) into acid form (poor water solubility). When APS was used as an oxidant and CTAB was used as a surfactant, PPy-NWs were formed (Figure 3e,f), mainly due to hydrophobic pyrrole monomer entered the micelles formed by CTAB with the cationic hydrophilic heads towards the outside, and after APS was added into the solution, a lamellar mesostructual (CTA)_2_S_2_O_8_ soft template was formed, and then pyrrole monomer was polymerized into PPy-NWs at the inward of the soft templates [15,28,29]. From the SEM and TEM images of PPy-NTs and PPy-NWs, we can see that the diameter of the prepared PPy-NTs and PPy-NWs are ~70 nm to 150 nm and ~30 nm to 60 nm, respectively, while the lengths of PPy-NTs and PPy-NWs are in the μm-range (Figure 3c–f). Based on the above-mentioned experimental results, we can conclude that the morphologies of PPy (e.g., PPy-PTs, PPy-NTs, and PPy-NWs) can be effectively controlled and tuned via adjusting the experimental conditions, such as adding or without adding different kinds of surfactants.

**Figure 4:**
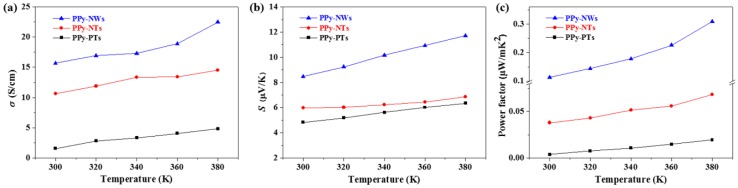
Electrical conductivity (**a**), Seebeck coefficient (**b**), and power factor (**c**), of the PPy-PTs, PPy-NTs, and PPy-NWs.

Figure 4 shows the electrical conductivity, Seebeck coefficient, and power factor of the PPy-PTs, PPy-NTs, and PPy-NWs. As the measured temperature increased from 300 to 380 K, both electrical conductivity and Seebeck coefficient of the PPy-PTs, PPy-NTs, and PPy-NWs increased, which leads to a same trend for the power factor. The electrical conductivity and Seebeck coefficient of PPy-NWs were much higher than those of PPy-PTs and PPy-NTs in the whole temperature ranges. This phenomenon was similar to that of PEDOT with different morphologies reported in Reference [12]. Considering the higher power factor of PPy-NWs, compared to PPy-PTs or PPy-NTs, hereafter, PPy-NW/graphene composites have been prepared and the influence of graphene contents on the morphologies and TE properties of the composites have been investigated and reported below.

**Figure 5:**
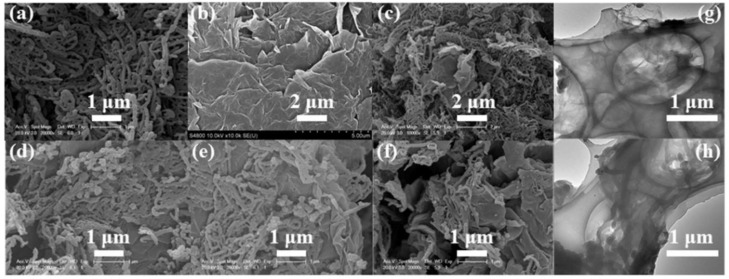
SEM images of (**a**) PPy-NWs, (**b**) graphene, PPy-NW/graphene composites with, (**c**) 5 wt %, (**d**) 10 wt %, (**e**) 15 wt %, and (**f**) 20 wt % of graphene. TEM images of (**g**) graphene and (**h**) PPy-NW/graphene composites with 20 wt % of graphene.

**Figure 6:**
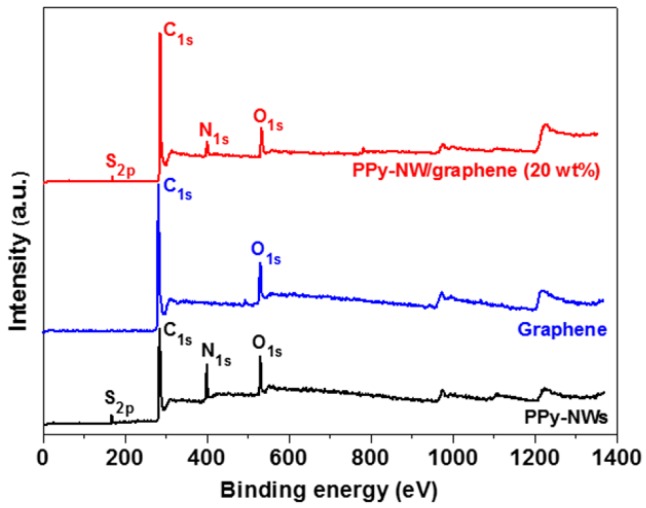
XPS spectra of wide region spectroscopy of graphene, PPy-NWs, and PPy-NW/graphene composites.

Figure 5 shows the SEM images of PPy-NWs, graphene and PPy-NW/graphene composites with different contents of graphene from 5 to 20 wt %, and TEM images of graphene and PPy-NW/graphene composites with 20 wt % graphene. The surfaces of PPy-NW/graphene composites (Figure 5c–f) were much rougher when compared to the pure graphene (Figure 5b), due to the surfaces of graphene were covered by PPy-NWs. It can be also clearly seen that as the content of graphene increased from 5 to 20 wt % (Figure 5c–f), the loading of PPy-NWs on the graphene surfaces decreased, mainly because of the surface areas of graphene increased in the nanocomposites. PPy-NWs have not only been uniformly coated on the surface of graphene, but also acted as bridges between different graphene (Figure 5c–f,h). The formation mechanism of PPy-NW/graphene composites is mainly due to the electrostatic attractions and π-π interactions between the graphene and hydrophobic pyrrole monomer [28,30], which made pyrrole monomer adsorbed on the surfaces of graphene. After APS was added into the solution, a lamellar mesostructual (CTA)_2_S_2_O_8_ soft template was formed, and then pyrrole monomer was polymerized into PPy-NWs at the inward of the soft templates and therefore adsorbed on the surfaces of graphene [15,28,29]. PPy-PTs and PPy-NTs mainly contain C, O, N, and Cl (Cl came from FeCl_3_·6H_2_O), while PPy-NWs and PPy-NW/graphene composites (Figure 6) have C, O, N, and S. It indicates PPy-NWs have been coated on the surface of graphene. Note that S comes from APS, which is agree with the Reference [31].

**Figure 7:**
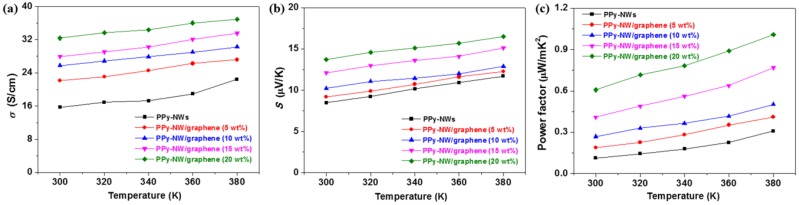
Electrical conductivity (**a**), Seebeck coefficient (**b**), and power factor (**c**), of PPy-NW/graphene composites with different content of graphene.

The electrical conductivity, Seebeck coefficient, and power factor of the PPy-NWs, and PPy-NW/graphene composites with different contents of graphene are shown in Figure 7. The electrical conductivity of the PPy-NW/graphene composites have increased as the content of graphene increasing from 0 to 20 wt %, e.g., from 15.7 S/cm for the pure PPy-NWs to 32.3 S/cm for the nanocomposites with 20 wt % graphene at 300 K. The reason for this phenomenon is mainly because of the following two aspects: (1) graphene has a much higher electrical conductivity than that of the PPy-NWs; and (2) PPy-NWs are not only coated on the surfaces of graphene, but also acted as bridges between graphene in the nanocomposites (see Figure 5), which benefits for the carriers’ transportation. As the content of graphene increases from 0 to 20 wt %, the Seebeck coefficient of the nanocomposites increases from 8.5 to 13.7 μV/K at 300 K, probably due to more interfaces have been introduced in the nanocomposites. The power factor of the nanocomposites increases from 0.1 μWm^−1^K^−2^ to 0.6 μWm^−1^K^−2^ with the contents of graphene increasing from 0 to 20 wt % at 300 K. It is mainly because of the simultaneous increase in conductivity and Seebeck coefficient of the nanocomposites. The electrical conductivity and Seebeck coefficient of the PPy-NW/graphene nanocomposites also increase slightly as the temperature increases from 300 to 380 K. E.g., the electrical conductivity and Seebeck coefficient increases from 32.3 S/cm (300 K) to 36.9 S/cm (380 K), and from 13.7 μV/K (300 K) to 16.5 μV/K (380 K), respectively, for the composites with 20 wt % of graphene. As a result, the power factor of the nanocomposites also increases as the temperature increases from 300 to 380 K, and a highest power factor of 1.01 μWm^−1^K^−2^ at 380 K has been obtained for the PPy-NW/graphene composites with 20 wt % of graphene. It is about 3.3 times higher than that pure PPy-NWs (0.31 μWm^−1^K^−2^ at 380 K). However, this value is still lower than that of a MWCNT/PPy composites (2.2 μWm^−1^K^−2^ at RT with 68 wt % MWCNT) [8], a SWCNT/PPy composites (5.46 μWm^−1^K^−2^ at 398 K with 40 wt % SWCNT) [9], or a rGO nanosheet/PPy composites (3.01 μWm^−1^K^−2^ with 66.7 wt % rGO at RT) [10]. It is mainly due to the contents of graphene in the studied nanocomposites (20 wt %) is much lower than that of MWCNT (68 wt %), SWCNT (40 wt %), or rGO (66.7 wt %) in the reported nanocomposites [8,9,10].

## 4. Conclusions

PPy with different morphologies (i.e., PPy-PTs, PPy-NTs, and PPy-NWs) have been successfully prepared and their TE properties have also been investigated. The results show that the PPy-NWs have the highest TE properties among all the morphologies of PPy. PPy-NW/graphene thermoelectric composites have been prepared via a soft template polymerization method. Both electrical conductivity and Seebeck of the PPy-NW/graphene composites increases along the contents of graphene from 0 to 20 wt %, or as the temperature increases from 300 K to 380 K. A highest power factor of 1.01 μWm^−1^K^−2^ at 380 K was obtained for the PPy-NW/graphene composites with 20 wt % of graphene. It was about 3.3 times higher than that of pure PPy-NWs.
